# Tools and approaches to ensure quality of vaccines throughout the cold chain

**DOI:** 10.1586/14760584.2014.923761

**Published:** 2014-05-28

**Authors:** Umit Kartoglu, Julie Milstien

**Affiliations:** ^a^ Department of Essential Medicines and Health Products, World Health Organization, 20 Avenue Appia, 27 Geneva 1211, Switzerland; ^b^ 3 bis rue des Coronilles, Résidence Parc de Clementville, Bâtiment C, 34070 Montpellier, France

**Keywords:** cold chain, cool water pack, supply chain, temperature monitoring device, temperature sensitivity, vaccine development, vaccine freezing, vaccine vial monitor

## Abstract

The Expanded Program on Immunization was designed 40 years ago for two types of vaccines: those that are heat stable but freeze sensitive and those that are stable to freezing but heat labile. A cold chain was developed for transport and storage of such vaccines and established in all countries, despite limited access to resources and electricity in the poorest areas. However, cold chain problems occur in all countries. Recent changes to vaccines and vaccine handling include development and introduction of new vaccines with a wide range of characteristics, improvement of heat stability of several basic vaccines, observation of vaccine freezing as a real threat, development of regulatory pathways for both vaccine development and the supply chain, and emergence of new temperature monitoring devices that can pinpoint and avoid problems. With such tools, public health groups have now encouraged development of vaccines labeled for use in flexible cold chains and these tools should be considered for future systems.

Immunization is one of the most successful and cost-effective health interventions known [1]. Over the past several decades, immunization has achieved eradication of smallpox [2], lowered the global incidence of polio by 99% [3], and reduced illness, disability and death from vaccine-preventable diseases. More children are being reached with vaccination (over 100 million per year in 2005–2007) [4]. With the addition of preventive campaigns to age groups at risk for meningitis A and yellow fever, access and use of vaccines by age groups other than infants is expanding [5].

Immunization is a key component behind the efforts taken to meet the Millennium Development Goals, particularly goal 4, which is reducing the child mortality rates [6]. For the first time, the number of children dying each year has fallen below 10 million as a result of the combined impact of improved access to water and sanitation, increased immunization coverage and integrated delivery of essential health interventions [7]. Part of this impact was due to the introduction of several new vaccines to most countries. Vaccines against hepatitis B and *Haemophilus influenzae* type b (Hib) have become part of the national immunization program in 179 countries [8]. Additional vaccines against priority diseases, such as pneumonia and diarrhea, have been introduced in many countries of the world [9,10] and others against chronic diseases such as cervical cancer are following.

More money is available for immunization through innovative financing mechanisms, including the Global Alliance for Vaccines and Immunization, other partnerships for specific disease control initiatives and the International Finance Facility for Immunization. In addition, countries are increasingly shouldering the burden for financing their own vaccines: 154 Member States of WHO report having a specific budget line for immunization and have developed multi-year action plans to sustain the gains achieved and further improve performance [11]. Such initiatives have supported global and regional immunization initiatives in low- and middle-income countries [12].

Despite this progress, vaccine-preventable diseases remain a major cause of morbidity and mortality [13]. Immunization coverage is not uniform from country to country or even within countries. The number of children unvaccinated with the six basic Expanded Program on Immunization (EPI) antigens declined only from 30 million in 2000 to 22.4 million in 2011 [14], which indicates that almost 20% of children born each year are still unreached. Moreover, roughly 1.6 billion people, or one-quarter of the global population, still have no access to electricity [15]. This has a direct impact on the access to immunization services which require cold chain storage and distribution of vaccines.

This paper will focus on two major aspects of immunization programs, their status and how they can be improved: tools and methods for improving access to vaccines by removing the obstacles in maintaining a refrigerated supply chain, and tools and methods for assuring the quality of vaccines throughout the delivery pathway. Part 2 explains the need for a ‘cold chain’ due to the inherent temperature sensitivity of vaccines. This section also explains that cold chain problems are global and have been documented in all countries where a temperature monitoring study has been conducted. Part 3 provides solutions that address both aspects mentioned above. Part 4 addresses the regulatory aspects of both improving the vaccine stability and assuring the quality of the supply chain. Part 5 summarizes the conclusions and outlines some future perspectives in these areas.

## The inherent temperature sensitivity of vaccines

Antigen instability is an inherent attribute of vaccines because of the complex nature of the three-dimensional structure of these biological polymers. Classically, there have been two general types of vaccines, live viral and bacterial vaccines, which do not require adjuvants to boost the immune responses but are more sensitive to potency loss during storage and distribution, especially at elevated temperatures, and non-replicating vaccines, such as inactivated viruses and bacteria, purified protein and carbohydrate antigens, which often require adjuvants to boost the immune responses. They are typically stable to moderate heat exposure, but mostly due to adjuvants, are sensitive to freezing. The current cold chain was developed for these two types of vaccines: those whose temperature sensitivity is intrinsic to the structure of the vaccine antigen and those whose temperature sensitivity is related to additives and adjuvants.

The live vaccines contain weakened, attenuated versions of infectious viruses and bacteria that can replicate *in vivo* (and, therefore, mimic natural infection). Live vaccines require careful maintenance of the vaccine cold chain. For example, the varicella-containing vaccines may even require frozen storage to ensure long stability, even in the lyophilized state, and thus can rapidly lose potency under refrigerated storage.

The second category, non-replicating vaccines (as they cannot replicate *in vivo*), usually require adjuvants in lieu of prohibitively high doses to provide sufficient levels of protective immunity in humans. From a stability viewpoint, inactivated and subunit vaccines are generally more stable and are typically available as liquid formulations.

These vaccines, however, can be freeze sensitive, especially if adjuvanted with aluminum salts, which may collapse on freezing, lowering the adjuvant effect [16]. A recent study in the United States found a surprisingly high number of accidental exposures to freezing temperatures for vaccine vials labeled for storage at 2–8°C due to inappropriate shipping or interim storage at storage at health centers [17,18]. Unintended freezing of aluminum-adjuvanted vaccines during transportation and distribution in the vaccine cold chain has been documented in many other countries, both developing and industrialized [19–21].

**Figure 1.**
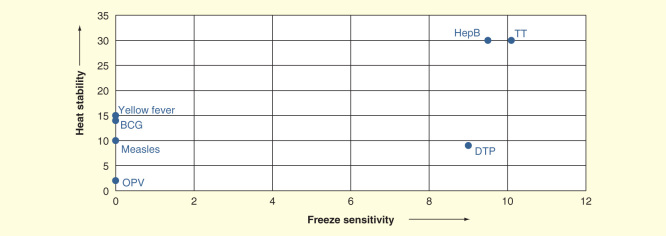
**Freeze sensitivity and heat stability of traditional vaccines.**

BCG: Bacille Carmen Guerin; DTP: Diphtheria–tetanus–pertussis; HepB: Hepatitis B; OPV: Oral poliomyelitis vaccine; TT: Tetanus toxoid.

**Figure 2.**
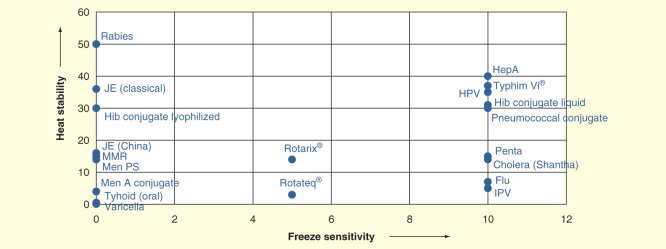
**Freeze sensitivity and heat stability of new vaccines.**

Many newer vaccines cannot be divided so easily into these two categories. Figures 1 & 2 show the breakdown, when freeze sensitivity is plotted versus heat stability for the traditional vaccines used in national immunization programs: oral poliomyelitis vaccine (OPV), measles, yellow feverBCG, tetanus toxoid, diphtheria–tetanus–pertussis and hepatitis B in Figure 1 and newer vaccines in Figure 2. In Figures 1 & 2, both axes are generated as ordinal scale based on the freezing sensitivity and heat stability of the mentioned vaccines. It can be readily seen that there are two major categories among the traditional vaccines, the live vaccines, which are not only freeze sensitive but are also not stable to high temperatures, and the inactivated vaccines, which cannot be frozen, but are heat stable. However, since 2000, new vaccines have been incorporated into national immunization programs, and more are on the threshold. These include those already incorporated, at least into some programs: measles–mumps–rubella, Hib conjugate, pentavalent vaccine, meningococcal, both polysaccharide and conjugate, rotavirus vaccines, pneumococcal conjugate, inactivated polio vaccine, human papilloma virus vaccine, rabies and influenza vaccines, and those on the threshold (Japanese encephalitis, hepatitis A, varicella, cholera and typhoid vaccines). Figure 2 shows a similar plot to Figure 1; there is a wider range of behaviors. There are vaccines that are freeze stable but range in heat stability from very low to very high, some with intermediate stability to freezing, a large number for which the manufacturers state they should not be frozen, and some with extremely high stability. Today’s cold chain must accommodate all of these products.

Hep A: Hepatitis A; Hib: *Haemophilus influenza* type b; HPV: Human papilloma virus; IPV: Inactivated polio vaccine; JE: Japanese encephalitis; Men A: Meningitis A; Men PS: Meningitis polysaccharide; MMR: Measles–mumps–rubella; Penta: DTP+HepB+Hib; Rotarix^®^ and Rotateq^®^: Rotavirus vaccine (GSK and Merck); Typhim Vi^®^: Typhoid polysaccharide vaccine (Sanofi Pasteur).

To ensure the optimal potency of vaccines, careful attention is needed in handling practices at all levels of the cold chain. These include storage and transport of vaccines from the manufacturer through the primary vaccine store down to the end user at the health facility and further down at the outreach sites. A cold chain is the integrated system of equipments (e.g., cold rooms, shipping containers, refrigerators, vehicles), procedures, records, and activities used to handle, store, transport, distribute and monitor temperature-sensitive products [22,23]. The allusion to a chain is apt. As with a physical chain, a cold chain is only as strong as its weakest link.

There is a general illusion that cold chain problems are mainly observed in developing countries. On the contrary, cold chain problems have been documented in all countries where a temperature monitoring study has been conducted. Many of these studies had been published, either as a report or in peer-review journals [24–28]. The Effective Vaccine Management initiative launched by WHO also documented poor compliance with temperature monitoring requirements at all levels of the vaccine cold chain in over 45 countries [Garnett A, Unpublished Data]. The most recent report on temperature violations comes from the US Department of Health and Human Services, Office of Inspector General [18]. In a study of 45 selected service providers, vaccines stored by 76% of the providers were exposed to inappropriate temperatures for at least five cumulative hours during the 2-week period. None of the 45 providers met the vaccine management requirements as established in the Vaccines for Children Program Operation Guide.

## Identified solutions

Currently, various solutions exist to ensure vaccine quality throughout the cold chain. Although all solutions explained below are available today, in a longer run, we expect more vaccines to become more stable which will have an impact on handing of vaccines through reducing the dependency on the cold chain. Controlled temperature chain (CTC) approaches can also be considered as long-term solutions since it requires a lengthy process of national regulatory authorities’ approval. In shorter term, the biggest challenge for all other available solutions is to incorporate them into immunization programs.

### Understanding the true stability of vaccines

Because the original problem with the cold chain was keeping the vaccines cold enough, most of the training efforts for the EPI were directed at that. It was not until the past 10–15 years that it was realized that vaccines were more heat stable than had been thought, and that freezing was more of a threat to vaccine integrity. There were three major events that led to this realization: improved heat stability of vaccines, stimulated in part by the new WHO guidelines requiring more stability, for example, for OPV [29], measles vaccine [30], and yellow fever vaccine [31], and in part by initiatives such as the Children’s Vaccine Initiative, which encouraged more stable vaccines; the development and use of vaccine vial monitors (VVMs) on vaccine provided through the United Nations Children’s Fund [32], which allowed health workers to see that vaccines could, in fact, stand exposure to temperatures above 8°C without losing significant potency and opened the way for their use in less-stringent temperature control situations [33–35]; the finding of tetanus disease in infants whose mothers had received sufficient and timely doses of tetanus toxoid, which led to the observation that instances of freezing of tetanus toxoid were common and led to lower potency vaccines [36,37], and additional publications which showed that vaccines were being systematically exposed to freezing temperatures in the cold chain [19–21,38]. These events required efforts to better educate health workers on the true stability of vaccines.

### Making vaccines more stable

Earlier efforts at increasing thermostability had been promoted by increasing the guidelines for vaccine thermostability in WHO vaccine quality guidelines, thus relying on vaccine manufacturers to do the requisite research. This approach has resulted in generally more stable vaccines. For example, at the time of the revision of the measles guidelines, not all measles vaccines being supplied in the public sector could meet the heightened thermostability requirements, but within a short period to time, most measles vaccines were in compliance. The past 20 years have seen increased stimulus by the public sector to fund some of the research in this area. As previously mentioned, the Children’s Vaccine Initiative aimed to increase vaccine thermostability, specifically starting with improving the thermostability of OPV. An initiative of program for appropriate technology in health has worked with vaccine manufacturers to follow-up promising leads to make vaccines more stable [39,40]. The work has already resulted in a more stable hepatitis B vaccine [40].

By focusing attention on vaccines that are in the late stages of development, not yet licensed for use, large public sector organizations that procure vaccines, such as the United Nations Children’s Fund, can encourage manufacturers to develop such vaccines, optimizing stability before pivotal clinical trials for licensure are done. Evidence suggests that manufacturers are beginning to recognize the advantages of documenting the stability of newer vaccines. Examples include the meningitis conjugate vaccines [41] and human papilloma virus vaccine [42]. The most useful innovations occur when the product insert mentions that the vaccines can be stored and used for limited periods of time at temperatures above the 2–8°C recommendations [Who Meeting in December 2012 in Ottawa, Canada on Scientific and Regulatory Considerations on the Stability Evaluation of Vaccines Under Ctc, Unpublished Data]. WHO is now conducting meetings with regulatory authorities to develop a consensus on this approach.

### Improving access to stability information

Once the stability is documented and the vaccine is relicensed with a new product insert, the next step is making sure that immunization managers and health workers have access to this information and are able to use it.

WHO makes some of this information available through monographs, such as the document, *Temperature sensitivity of vaccines* [43], through various guidelines for vaccine use [44,45], and the approved product insert for each WHO prequalified vaccine is now published on the prequalification website [46], which allows immunization managers to directly validate usage recommendations in accordance with their national needs. This provision of information has followed the publication of stability requirements as a basis for prequalification, in addition to the elaboration of target product profiles which give an indication of what temperature stability is desired. If a vaccine that is submitted for WHO prequalification does not meet these guidelines, the information is presented to the Programmatic Suitability for WHO Prequalification Standing Committee [47] for a recommendation as to whether the product should proceed through the prequalification process. In this way, WHO assures that products approved for UN agency purchase are optimally formulated for use in the field.

### WHO guidelines & directives to interpret stability information

WHO recommendations and guidelines for production and control of vaccines and other biologicals feature stability as a critical element and provide guidance for national regulatory authorities and vaccine manufacturers. The ‘Guidelines for Stability Evaluation of Vaccines’ were developed to complement current WHO recommendations for vaccine stability testing [48]. The guidelines aim to provide a scientific basis and guiding principles for evaluation of vaccine stability for the purpose of clinical trial approval, licensing and post-licensure monitoring. The first part of the new guidance document is devoted to general considerations on the stability evaluation of vaccines. This is followed by a discussion on the stability of vaccines during the manufacturing process and in subsequent use, focusing on intermediates and final products. Regulatory expectations for stability studies to be conducted at different stages of development are indicated in a separate section. The selection of samples and assays employed in the studies performed for different purposes, as well as the expression of results are discussed in the section on the design of stability studies and statistical considerations.

Key issues in the analysis of data are also considered and approaches to the analysis of the results of stability testing are described. Early in the development process, the manufacturer is encouraged to discuss approaches for the study design and data analysis and their suitability for the product in question with the national regulatory authority.

### Tools for the prevention of freezing

In a recent systematic literature review, the analysis highlighted that accidental freezing is pervasive and occurs across all segments of the cold chain. Between 14 and 35% of refrigerators or transport shipments were found to have exposed vaccines to freezing temperatures, while in studies that examined all segments of distribution, between 75 and 100% of the vaccine shipments were exposed [20].

Freezing of vaccines occurs when vials are exposed to temperatures below 0°C either during storage or transport, depending upon a host of factors, including the duration that the vaccine is exposed and whether the vaccine is agitated during that time period. From this perspective, we need to distinguish between ‘being exposed to freezing temperatures’ and ‘actual freezing’ [49]. In a study conducted in Thailand, vaccine shipments exposed to freezing temperatures were found to be unaffected by freezing [21]. The shake test is the only test with 100% sensitivity, 100% specificity and 100% positive predictive value to determine whether aluminum-adjuvanted freeze-sensitive vaccines have been affected by freezing. Although this test is not a tool to prevent freezing, it becomes an important tool to distinguish whether a freeze-sensitive vaccine has been affected by freezing, thus reducing wastage [50–53].

The best way to identify what needs to be done to prevent freezing is to apply risk assessment and management principles. Although risk assessment/management is a growing culture within the vaccine industry, there is little evidence that it is practiced by the public sector vaccine handling institutions. Risk assessment and risk management are used to make data-supported, proactive decisions on how to use resources to prevent occurrence of unwanted events (in our case, freezing), and should they occur, how to protect the assets of value in the environment [54–57]. In a typical risk assessment, one seeks answers to a set of risk questions through a system/process analysis using various tools [58–61]. Based on the particular circumstances of processes, the risk analysis results in grading the identified risks with ‘risk score’ and allows responsible staff to prioritize the control measures for introduction [57]. It is critical that public sector vaccine management institutions promote the risk management culture.

Although many technical interventions have been defined to prevent freezing of vaccines (e.g., no shelving within the plume of cold air close to evaporator and fitting mesh cage to prevent vaccines being placed within the danger zone in cold rooms) [62,63], in this paper, we will focus on three critical tools to prevent freezing of vaccines: improved temperature control, removal of ice from in-country transport, and use of VVM.

## Improved temperature control

In order to ensure quality and integrity of the vaccine products, their storage and distribution conditions should be monitored continuously. The general practice for temperature monitoring in vaccine refrigerators at the periphery is to use a thermometer (stem thermometer or bi-metal thermometer). A thermometer, however, only provides a snapshot of the temperature at the point in time when it is checked and cannot be considered as an ‘appropriate’ monitoring tool. If a temperature value of between +2°C and +8°C is found while checking, health workers may erroneously conclude that the vaccines are safe since this snapshot reading provides a value only when it is checked and does not cover the rest of the daytime/nighttime period. Unless a temperature excursion is seen at the time the temperature is checked with a regular thermometer, almost all temperature violations go unnoticed [64].

Different purpose-designed temperature monitoring devices are used at different levels of the cold chain. Table 1 illustrates WHO-recommended temperature monitoring devices throughout the cold chain [65].

**Table 1.** **WHO-recommended temperature monitoring devices for storage and transportation of vaccines.**

**Description**	**International transport**	**Primary vaccine store**	**Transport**	**Intermediate vaccine store**	**Transport**	**Service level**
Electronic shipping indicators	✓		✓			
Vaccine cold chain monitor	✓		✓			
Vaccine vial monitor	✓	✓	✓	✓	✓	✓
Irreversible freeze indicator	✓	✓	✓	✓	✓	✓
Programmable electronic temperature and event logger systems with integral alarm and auto-dialer options		✓		✓		
Integrated electronic maximum–minimum thermometer, with factory-programmed alarms, for vaccine refrigerators and freezers		✓		✓		✓
Wall-mounted pen recording thermometer		✓		✓		
User-programmable temperature data loggers	✓	✓	✓	✓	✓	✓
30-day electronic refrigerator temperature logger		✓		✓		✓

In a study to establish superiority of the 30-day electronic refrigerator temperature logger against thermometers, it was found that almost all temperature violations were missed in the thermometer group while all violations were caught by the 30-day device. The authors strongly recommended abandoning the use of thermometers as temperature monitoring devices for vaccine refrigerators and replacing them with 30-day electronic refrigerator temperature loggers [64]. Corrective actions to prevent temperature violations can be introduced only if such violations can be monitored throughout the storage period of vaccines.

## Removal of ice from in-country transport & introduction of cool water packs

**Figure 3.**
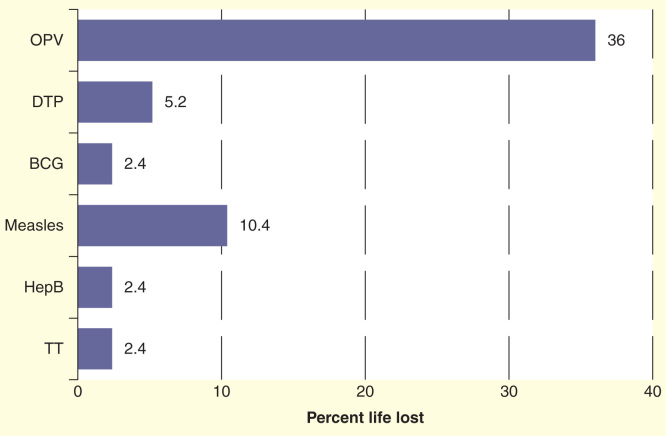
**Temperature impact on life loss of vaccines calculated on the basis of vaccine vial monitor reaction.** Each transportation is assumed to be done at a continuous temperature of +43°C for a period of 48 h with a minimum temperature reading inside the vaccine transport box recorded as 11.5°C, a maximum of 25.3°C and an average of 18.9°C throughout each journey. This scenario was repeated four times.

DTP: Diphtheria–tetanus–pertussis; HepB: Hepatitis B; OPV: Oral poliomyelitis vaccine; TT: Tetanus toxoid. Data taken from [49].

Presence of ‘ice’ is the main factor in exposing vaccines to freezing temperatures during transport. Insufficient isolation of the vaccines from the coolant and insufficient icepack conditioning are the main contributing factors to this. Between 2002 and 2004, WHO conducted a series of controlled laboratory studies and field tests (in Nepal, Myanmar, Turkey and Zimbabwe) to assess the impact of using cool water packs (pre-cooled to a temperature between +2°C and +8°C before use) on the cold life of vaccine transportation boxes and on the shelf life of the vaccines [49]. Evaluations were conducted to verify the assumption that cool water packs can safely replace the use of icepacks for the transport of vaccines and, at the same time, prevent the freezing of vaccines. Based on the recorded temperatures, the remaining VVM life of the vaccines was calculated using the Arrhenius equation. Based on the results, investigators defined ‘cool life’ (from +2 to +20°C) as a safety margin, such that all vaccines, except OPV, can safely be transported with cool water packs even in hot climates and up to a repetition of four times [49]. Figure 3 illustrates the impact of temperatures to vaccine shelf life calculated based on VVM reaction.

The presence of ice below 0°C is the main factor contributing to the freezing of vaccines during transport. The removal of ice eliminates the risk of vaccine freezing. The above results, however, demonstrate that the use of cool water packs is a safe practice for all vaccines except OPV. This clearly indicates that water packs can safely replace frozen icepacks without any damage to the vaccine potency or any major impact on vaccine shelf life. Successful implementation of this dual vaccine transport system (one for OPV and the other for all other vaccines) has been observed in Moldova during an assessment [Kartoglu U, Babalioglu N, Unpublished Data].

The new WHO Performance, Quality and Safety specifications bring clarity to the issue and recommend only cool water packs for freeze-sensitive vaccines [66,67]. The new specifications also redefine cold life, cool life and warm life as performance indicators for the insulated containers, cold boxes and vaccine carriers.

## Vaccine vial monitors

The VVM is the only tool among all time–temperature indicators that is available at any time in the process of distribution and at the time a vaccine is administered, indicating whether the vaccine has been exposed to a combination of excessive temperature over time and whether it is likely to have been damaged. It clearly indicates whether a vaccine can be used [68]. Although developed as a heat-exposure indicator, VVM also contributes significantly to the reduction of vaccine freezing since it allows health workers to see the heat stability of vaccines and accept the fact that freezing is a greater danger than mild heat exposure [68–71]. In this regard, WHO recommends all Member States to consider adoption of policies permitting the use of vaccines beyond the cold chain where warranted for routine immunization activities or on a limited basis in certain areas or under special circumstances, such as the following [32,45]:

- National immunization days;
- Hard-to-reach geographic areas;
- Immunizations provided at home, including hepatitis B vaccine birth dose;
- Cool seasons and;
- Storage and transportation of freeze-sensitive vaccines (diphtheria-tetanus-pertussis, tetanus toxoid, diphtheria-tetanus, tetanus and diphtheria (reduced component) toxoid, hepatitis B and Hib vaccines) where the risk of freezing is greater than the risk of heat exposure.

Adoption of such policies becomes even more critical in places where great proportions of the population do not have access to electricity. More than 99% of the 1.6 billion people without electricity live in developing regions of the world, and four out of five live in rural areas of South Asia and sub-Saharan Africa. Under the status quo and absent radically new innovations in global energy policy, by 2030, it is estimated that 1.4 billion people still will not have access to electricity [72].

### Studies using vaccines outside of the traditional cold chain

WHO has been exploring the possibilities of loosening the strictures of the traditional cold chain for many years, but fear of losing the discipline and monitoring that has accompanied the cold chain has hampered progress in this direction, along with the low number of vaccines with label indications documenting their enhanced stability. Now, however, such vaccines exist, and thus Project Optimize has recently defined a CTC [73,74], whereby specific vaccines are prequalified for use at temperatures of up to 40°C for limited periods of time as appropriate to the stability of the antigen. For vaccines authorized for use in a CTC, specific guidance on how to maintain the correct temperature range and adhere to the other pre-defined CTC conditions is available from WHO.

The CTC has been defined through a series of meetings convened by Project Optimize and by WHO [75]. The key criteria [agreed at a meeting in 2012 of a WHO drafting group on stability evaluation for the CTC, Unpublished Data] are that the vaccine is being used in accordance with the product package insert, that the vaccines are not OUT of the cold chain and are still under control, but the temperatures of storage and recommendations for CTC use of products are for products prequalified by WHO, produced by International Federation of Pharmaceutical Manufacturers & Associations manufacturers and with a public health benefit. However, the first vaccine approved for CTC use is MenAfriVac^®^, a conjugate meningitis vaccine developed and produced by the Serum Institute of India for use in the African meningitis belt, and this approach has recently been field-tested in Benin [76,77].

### Human resources

In order to realize the above-listed approaches, responsible staff at all levels must have necessary knowledge and skills [78]. Vaccine cold chain is no longer simple, as the new vaccines are added to the immunization schedules and responsible staff must be trained on different handling requirements. Training is also needed on temperature monitoring through new devices including electronic temperature recording monitors and VVMs, as well as the use of cool water packs. Many of the solutions presented require using new management methods, especially risk management approaches. Managers must learn how to apply risk analysis in all processes that affect vaccine quality, so that they can develop prioritized preventive, detective and mitigation measures. In addition to classical classroom style vaccine management courses, WHO has developed two authentic e-learning courses to support this: pharmaceutical cold chain management and VVM-based vaccine management [79,80].

## Regulatory aspects of solutions

### Regulation of the supply chain

Public and industry members of the vaccine cold chain have various global regulatory requirements to meet while handling, storing and distributing temperature-sensitive products to ensure that the quality and efficacy of the product will not be compromised [81]. To this effect, different bodies throughout the world issue regulatory oversight documents for guidance. Three type of institutions can be counted in issuance of such documents: national regulatory agencies; other regulatory bodies, for example, the International Air Transport Association (IATA) [82] and the International Conference on Harmonization [83]; and international organizations, for example, WHO [84] and the Parenteral Drug Association [23].

National regulatory agencies of Member States (e.g., USFDA, Health Canada [85], EMA [86]) issue guidelines for temperature control of drug products during storage and transportation. Recently, with the increased importance of pharmaceutical cold chain management, major revisions were done to these guidelines including the ones from the United States Pharmacopeia, Health Canada and the European Union. With the revised documents <1079> *Good Storage and Distribution Practices for Drug Products* [87], <1118> *Monitoring Devices – Time, Temperature and* Humidity [88] and the brand new <1083> *Good Distribution Practices – Supply Chain Integrity* [89], the United States Pharmacopeia announced significant changes. However, it should be noted that all these documents are ‘guidelines’ in nature, putting forward principles to set standards or determine a course of action. Although there is no obligation by law for the industry to follow these guidelines, it is in their interest to do so. One other detail in these guidelines is that they describe ‘what’ needs to be done; ‘how’ this could be done is not explained. In this regard, the move from WHO to publish 16 technical supplements on best practices to support the harmonized ‘Model guidance for the storage and transport of time- and temperature-sensitive pharmaceutical products’ can be mentioned as an important development [84].

International conference on harmonization guidelines have been adopted as law in several countries, but mainly used as the guidance in many. IATA recently published new regulations for airlines, ‘Air transport logistics for time- and temperature-sensitive healthcare products’, otherwise known as IATA Chapter 17 of the Perishable Cargo Regulations, which is now in effect [90]. With this new regulation, IATA also harmonizes the label to go on all time- and temperature-sensitive products for air transport. The extensive revisions offer a holistic approach to handling and the distribution of supply chain, with more practical, more relevant and clearer requirements than previous editions.

All regulatory guidance documents intend to provide general guidance concerning storage, distribution and shipping of pharmacopeial preparations and describe procedures to maintain proper storage environments for individual products and to ensure products’ integrity until it reaches the end user. From this perspective, it can be concluded that the regulatory trend today is for increased oversight, management and control of environmental conditions across the entire supply chain from manufacturer to consumer for temperature-sensitive pharmaceutical products. In this regard, more pressure is now on the public sector handling vaccines after their arrival to the country.

### Regulatory issues related to vaccine quality

Regulation of vaccines is complex and multifactorial, including, first of all, good control of the process and independent oversight of production and testing by a competent regulatory authority. Regulatory activities start at the very beginning of vaccine development and continue throughout its life cycle [91].

WHO has developed a guideline on the evaluation of vaccine temperature stability by testing [48]. The goals of stability studies include the establishment of product characteristics to support the proposed shelf life for determining the expiration date and to support changes in product manufacture, including improvements in vaccine stability [77]. Stability testing will be done prior to licensing to establish a stability profile, as a release test as part of the overall package and post-licensing to detect anomalies in the product integrity. There are a number of important considerations that are outlined in this unpublished report:

- The characteristics of the quality being tested should be reflected consistently in the type of test done. For example, loss of potency, as measured by a validated potency test, should reflect the loss of vaccine efficacy. Other characteristics that could correlate with vaccine integrity can be measured instead if correlated with vaccine efficacy. Often a battery of tests might be needed to give a complete stability picture.
- Stability studies necessary to support product licensure or a change to a license may not be sufficiently rigorous to mimic real-life situations. Current models do not allow for unplanned temperature excursions, and thus, the approach is to define limited conditions for planned temperature excursions (such as CTC use) and then assure a ‘stability reserve’.
- Correlation with VVMs or other time–temperature indicators: In general, this will depend on the fact that the product follows Arrhenius kinetics of inactivation or that the time–temperature indicators reach the end point before the product reaches label claim in the relevant temperature range.

In addition, just knowing the vaccine stability will not be sufficient for using the vaccine in a CTC, with defined temperature excursions. The temperature range and time period must be controlled, including whether or not the product will be returned to the cold chain, where it is in its dating period, and the impact of stabilizers. However, it is important that vaccines be used according to the information in the approved product insert, as this is a legal document, and using the product ‘off-label’ implies that the vaccine administrator takes on the liability.

There are several examples of vaccines already in the public domain where label changes have been approved for temperature excursions. In the United States, both the currently available human papillomavirus vaccines have such approvals: Gardasil^®^ can be exposed to temperatures at or below 25°C for 72 h and Cervarix^®^ can be stored for 3 days between 20 and 25°C or for 1 day between 25°C and 37°C. The latter variation to the product insert has not been approved by WHO for the vaccine as prequalified because it does not correlate with VVM characteristics. MenAfriVac has been approved for up to 4 days storage at 40°C immediately before use. BioManguinhos and Sanofi Pasteur have reported ongoing studies to develop label changes for storage and use of yellow fever vaccines and diluent for limited time periods up to 40°C.

## Expert commentary & five-year view

When the management and infrastructure of the EPI were being established, there was no way for healthcare providers in the system to determine if the vials of vaccines were spoiled as a result of the cumulative effects of heat exposure along their years-long journey. With the limited number of antigens in the EPI, cold chain systems were governed through a single set of rules without taking into account the local environments, which led to the gradual emergence of a dogmatic view of the cold chain. As a result, the cold chain became too cold. Although the number of antigens in the EPI was very limited, there were some vaccines with quite high heat stability. Health workers could have used this information to full advantage to increase access to remote populations.

Over the past years, immunization programs have evolved and diversified, and many more new vaccines have been added to the program, with quite different heat stability profiles requiring a different approach from those of the past EPI. Moreover, vaccines have become more stable. Public sector organizations that procure vaccines are increasingly demanding newer vaccines that are more stable and encouraging manufacturers to develop such vaccines. Public sector organizations are also encouraging manufacturers to relicense products with certain field advantages to take them outside the 2–8°C temperature cage. To this effect, national regulatory authorities are incorporating the most recent developments in good storage and good distribution practices into their regulatory oversight documents and issuing new ones if necessary.

The new approaches such as removal of ice from in-country transport and introducing the use of cool water packs and VVMs, and improved temperature monitoring tools, and new vaccine management modalities now make populations more reachable. The new definition of ‘cool life’ to allow vaccines being transported with cool water packs will also be one of the revolutionary changes in classic cold chain policies. Today, the VVM can be seen as a catalyst for much-needed changes in the strategies of vaccine distribution via the cold chain. It should eventually allow immunization programs to exploit the stability of each vaccine to the greatest possible extent, minimize distribution costs and increase flexibility in the handling of vaccines in the field, thus helping to make operations more effective.

Although there is a trend in making vaccine stability information available for health professionals, without tools like the VVM, it will be difficult to use this information to its utmost potential since violations along the supply chain will not be recorded and passed on to the new users.

The future of the cold chain will also be shaped with new technologies such as improved passive storage devices, new insulation technologies and super-efficient compressors, and improved temperature controls [92]. Improved electronic recording thermometers that are affordable at the health center level will also help in improving the quality of the cold chain. Through such devices, health workers and supervisors will feel more comfortable in knowing all the temperature exposures (especially low alarms) during long weekends and holidays.

Risk assessment and risk management culture should be adopted by all public and private immunization sectors to identify and introduce prioritized control measures to prevent and mitigate unwanted events.

Vaccine distribution without a cold chain would considerably simplify the delivery system and make it easier to integrate with drug distribution in countries.

## Notes

Key issues

- In the past almost 40 years, Expanded Program on Immunization (EPI) has been one of the most successful and cost-effective interventions known in terms of saving the lives of infants and their mothers. This progress has been made despite the fact that most vaccines need some kind of cold chain, and about one-fourth of the global population still has no access to electricity and four-fifths of these live in rural areas of South Asia and sub-Saharan Africa.
- The EPI vaccine delivery system has been based on vaccines that fall into two categories: those that are heat sensitive but stable to freezing and those that are stable for days at temperatures up to 40°C but sensitive to freezing. But newer vaccines numbering >30 have a wide range of temperature stability and sensitivity characteristics.
- One approach of improving the integrity of the vaccine delivery system is to remove the obstacles in maintaining a refrigerated supply chain, which can be approached by a combination of three methods: developing ways to prevent vaccine freezing, for example, by using cold water packs instead of ice for transport; using more effective time–temperature monitors that can go on every vial of vaccine from the time it leaves the manufacturer until it reaches the recipient; and by better control and regulation of the supply chain.
- A second approach is by using vaccines with improved stability to temperature variations and making this information widely known, which requires collaborations with vaccine manufacturers and regulators.
- Vaccines are now more heat stable than they were originally and some can now be safely used in a controlled temperature chain, with a relaxation of the strict 2–8°C standard previously used.
- Optimal immunization programs depend on a mix of stable vaccines used optimally in accordance with their stability, fitted with appropriate temperature monitoring devices on each vial, and by assessing the potential threat of temperature excursions using a risk management approach.
